# High-Strength and Heat-Insulating Cellular Building Concrete Based on Calcined Gypsum

**DOI:** 10.3390/ma16010118

**Published:** 2022-12-22

**Authors:** Adrian Ioana, Lucian Paunescu, Nicolae Constantin, Valeriu Rucai, Cristian Dobrescu, Vili Pasare, Alexandra Istrate

**Affiliations:** 1 Engineering and Management of Metallic Materials Obtaining Department, Science and Engineering Materials Faculty, University Politehnica of Bucharest, 060042 Bucharest, Romania; 2 Cosfel Actual SRL Bucharest, 021619 Bucharest, Romania; 3 Materials Science Department, Science and Engineering Materials Faculty, University Politehnica of Bucharest, 060042 Bucharest, Romania

**Keywords:** cellular concrete, aluminum powder, calcined gypsum, lightweight, mechanical strength

## Abstract

A cellular concrete with a fine porous structure was experimentally made using the corrosion technique for aluminum powder as an expanding agent in an aqueous solution of Ca(OH)_2_. The originality of this paper was the use of our own production method for the fine aluminum powder through atomizing the recycled molten waste of this metal using concentrated jets of nitrogen. Additionally, the waste melting technique involved our own microwave heating method. A high weight proportion of calcined gypsum (maximum 82.3%) represented the main concrete binder. Using moderate contents of coal fly ash (3.6–11.1%) together with perlite (4.6–6.4%) to reduce the pore size and silica fume (0.3–1.2%) with pozzolanic properties, the aim was to obtain a macrostructure characterized by a very low pore size and to increase the compressive strength (by up to 4.1 MPa), despite the relatively low density (below 641 kg/m^3^). An industrial method of increasing the mechanical strength by steam curing fresh concrete was applied.

## 1. Introduction

Cellular (aerated) concrete as a construction material has been designed and industrially manufactured since the first decades of the 20th century. The Swedish architect Axel Eriksson patented the production technique for this concrete type by mixing limestone and ground slate [1] in terms of its chemistry, the used mixture includes cement, lime, gypsum (anhydrous calcium sulfate), sand, and aluminum powder. Practically, the aggregate is not part of its composition. The role of the aluminum powder is important in the expansion process of the material mixture. Unlike other concrete types, the aerated concrete does not contain aggregate. The aluminum powder reacts with finely ground sand or fly ash, forming a homogeneous structure containing small hydrogen bubbles. Thus, the hardened concrete becomes a porous and lightweight material. Since 1880, it had been known that wet foamed materials can be quickly hardened using steam under pressure (known as the autoclaving process), and the shrinkage is almost zero after steam curing compared to normal air curing. Later, it was discovered that alternative materials such as fly ash could partially replace lime or cement, leading to savings in the use of the binder as an expensive raw material. Towards the end of the last century, autoclaved aerated concrete (AAC) began to be manufactured on a large scale in Germany, UK, Sweden, Denmark, and the Netherlands in blocks, panels, and reinforced elements. Several industrial plants were developed in the 1990s in Asia, Central Europe, and Eastern Europe [1].

In another study [2], particleboards containing waste rubber (tires and mixtures of isolators and carpets) filler were evaluated from the point of view of flammability. The assessment of the utilization of these composites in the construction industry was performed through the determination of their spontaneous ignition temperatures, mass burning rate, and calorific value. Based on the results for the spontaneous ignition temperatures, similar values between conventional particleboards and particleboards containing 10%, 15%, and 20% of waste tires were obtained. The average times ranged from 298 s to 309 s and the average temperatures from 428.1 °C to 431.7 °C. For the mass burning rate, there were similar results between conventional particleboards and particleboards containing 10% of waste tires and waste rubber. The time to initiation was 34 s and the time range to reaching a maximal burning rate was from 6 6 s to 68 s. The calorimetry results showed similar properties for the calorimetric values and ash contents in conventional particleboards and particleboards containing 10% of waste tires and waste rubber. The calorific value range was from 18.4 MJ·kg−1 to 19.7 MJ·kg−1 and the ash contents ranged from 0.5% to 2.9%.

In [3], the functional polyethers of N-(2,3-epoxypropyl)-4,5,6,7-tetrahydroindole (up to 61% yield, Mw = 8.7–11.7 kDa) and copolymers with ethylene glycol methylglycidyl ether (Mw = 5.6–14.2 kDa) and ethylene glycol vinylglycidyl ether (Mw = 6.4–12.3 kDa) were synthesized via anionic ring-opening polymerization in the presence of KOH without solvent. The polymerization involved the opening of the epoxy ring to deliver the linear polyethers bearing free tetrahydroindole rings and oxyethylene or vinyloxy groups in the side chain. The polyethers are soluble in ethanol, benzene, chloroform, dioxane, DMF, and DMSO. The obtained polyethers exhibited the properties of high-resistance organic semiconductors; their electrical conductivity range was 10∓14 S/cm.

The application of aerated concrete in construction has highlighted several advantages compared to common concrete, as it can maintain a comfortable climate in the building while reducing the heating cooling costs, it is fire-resistant, it shows resistance to earthquake activities, it is environmentally sustainability, and it provides effective insulation for sound [3,4].

Cellular concretes are considered as synthetic materials, with the hardened binder having a homogeneously distributed porous structure [5]. According to the type of binding agent, cellular concretes are based on cement (foam or aerated concrete), lime binder (foam silicate), and gypsum (foamed gypsum). In addition, gypsum–cement–pozzolanic binder mixtures and Portland cement–quicklime mixtures can be achieved. Additionally, cellular concretes can be made by adding previously prepared foam or by introducing foaming agents into the mixture (synthetic and protein foaming agents). The main properties of cellular concrete are its density (200–1200 kg/m^3^), thermal conductivity (0.06–0.40 W/mK), and compressive strength (0.4–15 MPa). The compressive strength is strongly influenced by the curing conditions. Autoclaved concretes (cured with steam at high pressure) have higher mechanical strength compared to concretes with common binder and common curing conditions.

Currently, there are several known manufacturing variants for aerated concrete, depending on the nature of the binding component (limestone, cement, limestone–cement mixtures, pulverized ash, granulated slag) and the type of siliceous component (natural materials such as silica flour sand and other sand types or industrial by-products such as coal fly ash, ore processing by-products, ferroalloy waste), as well as the curing conditions (autoclave hardening in a saturated steam medium above the atmospheric pressure and non-autoclave hardening under natural conditions through electrical heating in a saturated steam medium at the atmospheric pressure) [6].

The experimental production of non-autoclaved aerated concrete with mineral additives was tested according to [7]. Portland cement clinker (as an intermediate product in Portland cement manufacturing), quartz sand (80–90% quartz and 19–20% feldspar), and lump lime containing at least 80 wt.% CaO and MgO were used as the raw materials, along with mineral additives (5% diopside containing 56.1% SiO_2_ and 25.4% CaO or 7% wollastonite containing 53.4% SiO_2_ and 34.7% CaO). The choice of mineral additives took into account their influence on the hydration of the clinker minerals and the formation of the hardened cement paste structure. The characteristics of the experimentally manufactured concretes were density values of 580 kg/m^3^ (in the case of using diopside) and 600 kg/m^3^ (in the case of using wollastonite), compressive strength values of 3.3 MPa (in the case of using diopside) and 3.1 MPa (in the case of using wollastonite), as well as the thermal conductivity values of 0.131 W/mK (diopside) and 0.135 W/mK (wollastonite).

The use of fine siliceous sand as the aggregate and granulated blast furnace slag at a proportion of 50% as a partial substitute for cement, together with a soluble salt of polymeric sulfonates of organic compounds added into the mixture (between 0.2–2%) of the total cementitious material, contributed to increasing the compressive strength of the cellular concrete up to 9.5 MPa (after 7 days), but the concrete density was quite high (around 1300 kg/m^3^) [8].

ECOCON concrete is a type of lightweight aerated cellular concrete industrially manufactured as a dry powder mixture, which can be turned into a lightweight concrete by mixing it with water [9]. It is made using a unique manufacturing process with the application of nanotechnology, which allows the pre-design of the consistency of the concrete, with its final quality being independent of the production method (in-plant or in situ). ECOCON blocks exhibit several properties that contribute to their performance (weight range of 400–800 kg/m^3^, thermal and acoustic insulation, fireproof, excellent workability, high durability). ECOCON is an efficient and economic substitute for lightweight aggregate concrete, autoclaved aerated concrete (AAC), and sandwich blocks.

The phosphogypsum-based preparation technique for non-autoclaved aerated concrete was presented in [10]. The optimal concrete manufacturing recipe included 15% cement, 30% granulated blast furnace slag, 55% phosphogypsum, 7% quicklime, 1.6% sodium sulfate (Na_2_SO_4_), and 0.074% aluminum powder. The water/solid weight ratio was 0.45. The optimal steam temperature for concrete curing was 90 ºC. The main characteristics of the hardened monolithic material were a density of 690 kg/m^3^ and compressive strength of 3.5 MPa. The results demonstrated that the role of the phosphogypsum is not only as a filler, but also as an activator of the process. Experimentally, the aluminum powder contents varied in the range of 0.05–0.10%, contributing to reducing the concrete density from 830 to 600 kg/m^3^. The quicklime content range was 3–15%, modifying the density within low limits (665–690 kg/m^3^) but influencing the compressive strength, which reached the highest value of 3.5 MPa at 7–12% and showed lower values (2.8–3 MPa) outside this range. The Na_2_SO_4_ content of 1.2% allowed a decrease in concrete density up to 580 kg/m^3^ and influenced the increase in compressive strength from 5 MPa up to 6.4 MPa, corresponding to the proportion of 1.6%.

According to [11], phosphogypsum as an industrial by-product of the phosphoric acid manufacturing process has a major influence on the compressive strength and setting time of concrete, so it is suitable for use in the manufacturing process of non-autoclaved aerated concrete. Using the Taguchi method, the most influential factors (composition of Portland cement, phosphogypsum, and quicklime) that determine the characteristics of concrete had an important effect on the compressive strength of the aerated concrete. Thus, the optimal proportions of the raw material components used to obtain the compressive strength of 2.09 MPa and density of 806 kg/m^3^ were 34% Portland cement, 35% phosphogypsum, and 10% quicklime.

The other information from the literature [12,13,14] refers to the experimental results when making non-autoclaved aerated concrete. The powder mixture representing the binding material was composed of Portland cement (30%), gypsum-containing components (30%), and ash (40%). Aluminum powder was used in the amount of 6.55 kg/m^3^ and the ash was dosed at 309 kg/m^3^. The amount of working water was 371 kg/m^3^, with the water/solid ratio being 0.74. The steam temperature for concrete hardening was within the limits of 34–36 °C. The concrete specimens had the following characteristics after steam curing: density values in the range of 580–950 kg/m^3^, thermal conductivity values in the range of 0.17–0.24 W/mK, compressive strength values in the range of 3–8 MPa, and a frost resistance coefficient after 50 freeze–thaw cycles of 0.86.

The authors of the current work previously experimentally tested making cellular glass by releasing hydrogen as an expansion agent of the raw material through the corrosion of fine aluminum powder in the aqueous solution of calcium hydroxide, Ca(OH)_2_ [15]. The method substitutes the common types of foaming agents, with the gas released being hydrogen as a bubble former in the glass mass at room temperature. The cellular glass had excellent thermal insulation properties and acceptable compressive strength.

The same research team recently tested making a monolithic building material based on gypsum using aluminum powder in Ca(OH)_2_ solution, releasing hydrogen as an expansion agent of the raw material containing preponderantly gypsum [16]. The composition of the mixture used in that experiment included calcined gypsum (CaSO_4_·0.5H_2_O) below 100 μm, hydrated lime Ca(OH)_2_, coal fly ash below 80 μm, perlite as a light aggregate below 25 μm, silica fume as an effective pozzolanic material below 10 μm, carboxymethyl cellulose as a binder and foam stabilizer, as well as aluminum powder below 10 μm. The weight proportions of the composition were: 70.7–78.8% calcined gypsum, 9.5–10.4% Ca(OH)_2_, 3.4–5.1% coal fly ash, less than 10% perlite, 0.7–1.4% silica fume, 2% carboxymethyl cellulose, 3% aluminum powder, and 25–35% distilled water. The features of the cellular concrete were: density values of 530–600 kg/m^3^, porosity values of 71.4–74.7%, thermal conductivity values of 0.129–0.184 W/mK, compressive strength values of 1.2–2.2 MPa, water absorption values of 3.5–3.9 vol.%, and pore sizes below 4.5 mm. Lightweight gypsum materials can be used as thermal insulation blocks, boards, partition blocks, thermal insulation plasters, and fire-resistant plasters.

Considering that generating large pores plays a negative role in the stability of porous structures in cellular concrete and influences the compressive strength. which decreases, the aim of the research presented in the current paper was to make low-porosity cellular concrete that favors good correlations between the density, thermal conductivity, and compressive strength. The mixture expansion method involves corrosion reactions of fine aluminum powder in an aqueous solution of hydrated lime–Ca(OH)_2_, with the release of gaseous hydrogen also applied in the last experiment presented in [17]. The originality of the study was primarily based on the fine pulverization of molten aluminum waste in the microwave range using nitrogen. 

## 2. Materials and Methods

### 2.1. Methods

The gypsum commonly used in construction is semihydrated gypsum (CaSO_4_·0.5H_2_O), known as calcined gypsum. Calcined gypsum is considered one of the binders with adequate physical, thermal, and ecological properties for building concretes [18]. However, the specific weight range is quite high (60–80 kg/m^3^) [19], and it must be reduced with the addition of an inorganic filler (e.g., perlite, an amorphous volcanic glass that homogenizes the concrete macrostructure and significantly reduces the pore size) [20].

Silica fume (or microsilica) is an amorphous polymorph of silica dioxide (SiO_2_). It is an ultrafine powder (with less than 1 μm spheres) by-product of the industrial manufacture of silicon and ferrosilicon alloy. The main feature of silica fume is its pozzolanic property for high-performance concrete manufacturing. The addition of silica fume to the binder of the concrete leads to increased compressive strength, bond strength, and abrasion resistance [21]. 

Lime is added to the mixture as hydrated lime–Ca(OH)_2_ in the form of colorless crystals or white powder. Traditionally, Ca(OH)_2_ is obtained following the reaction between the quicklime (CaO) and water. Through its reaction with silica fume as a highly active pozzolana, the pozzolanic binder paste is hardened [22].

It is well known from the literature [23] that the addition of coal fly ash (by-product of coal burning in thermal power stations captured in electrofilters) to Portland cement concrete increases its performance, including its workability, mechanical strength, and durability, providing low proportion of interconnected pores in the concrete macrostructure and decreasing the permeability. However, in the current experiment, the proportion of fly ash was limited below 11 wt.% in order to obtain small and uniform pore sizes for the cellular concrete with moderate expansion by foaming the mixture. To achieve a particular fineness of the ash (below 44 μm), advanced mechanical processing was required.

The sodium salt of carboxymethyl cellulose (CMC), an anionic cellulose ether in powder form that is soluble in cold water, was also used in the starting mixture as a known foam stabilizer [24].

As mentioned above, the main component for the concrete manufacturing process is the very fine aluminum powder that is used as the gas (hydrogen)-supplying agent for the mixture. The method of releasing and blocking gaseous hydrogen in the mixture with the formation of bubbles is based on chemical reactions involving the corrosion of the surface of the fine aluminum powder in the aqueous medium containing Ca(OH)_2_.

According to [10], in which this method was developed with the objective of producing hydrogen for energy purposes, the aqueous solution of ionized Ca(OH)_2_ in the presence of the NaOH catalyst contains Ca^2+^ and OH^−^ ions and acts on the oxidized peripheral layer of aluminum particles. turning Al_2_O_3_ into aluminum hydroxide anions, which react with Ca^2+^, molecular-water-forming katoite [Ca_3_Al_2_(OH)_12_], and molecular hydrogen. The process took place at room temperature. Theoretically, the general reaction that characterizes the process of hydrogen release together with the solid phase, called katoite (which enters the molten mass), using aluminum powder in an aqueous solution of Ca(OH)_2_ is as follows:

2Al + 3Ca(OH)_2_ + 6H_2_O = Ca_3_Al_2_(OH)_12_ + 3H_2_ (1)

Practically, this reaction is more complicated, taking place in several stages and involving the participation of Ca^2+^ and OH^−^ ions [10].

A similar foaming method for a glass-waste-based mixture [25] was applied by the authors of the current paper at room temperature, obtaining cellular glass products with remarkable energy efficiency.

The main original element of the work was the production of a very fine non-oxidized aluminum powder in a plant using recycled aluminum waste as the raw material and concentric jets of gaseous nitrogen as the spraying agents, which were focused on the central jet of molten aluminum. Capturing the powder in an enclosure with cooled walls allowed very fine particles (below 10 μm) to be obtained without sticking occurring between them.

One modern industrial technique for improving the performance of cellular concrete (compressive strength, thermal conductivity, density, etc.) involves steam curing in autoclaves between 40 and 100 °C and at atmospheric pressure (in the case of non-autoclaved aerated concrete) [26] or at high pressure (in the case of autoclaved aerated concrete (AAC)). The experiment described in this paper aimed to approach the industrial conditions mentioned above by introducing the fresh concrete specimens in molds into a small, sealed enclosure that was constantly electrically heated at 80 ºC, inside which a flow of steam at 0.3 bar and 75 °C was periodically blown. The total duration that the fresh concrete was kept in the hot room for curing was 8 h, of which the intermittent steam blowing occurred for 3.5 h.

### 2.2. Materials

The solid materials used in the experiment and noted above were calcined gypsum (CaSO_4_·0.5H_2_O), hydrated lime [Ca(OH)_2_], coal fly ash, perlite, silica fume, CMC, and aluminum powder. The oxide compositions of coal fly ash, perlite, and silica fume are shown in Table 1.

**Figure 1:**
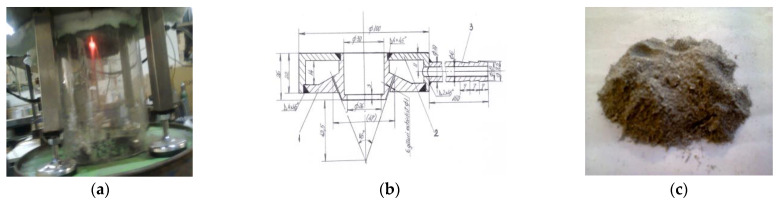
The details of the atomization of molten aluminum: (**a**,**b**) the atomization area of the installation; (**c**) the batch of aluminum powder (reproduced with permission from Junkoeko SRL Slobozia, Romania).

The very fine aluminum powder (under 10 μm) used in the experiment was produced by the team of authors at the Romanian companies Cosfel Actual and Daily Sourcing and Research using an original installation for molten metal atomization (Figure 1). The coarse ground waste was melted into a silicon carbide crucible, making the microwave irradiation more effective compared to the conventional heating techniques. The molten aluminum central jet was pulverized into very fine particles via the direct contact with the nitrogen jets used as the atomization agents. The solid aluminum particles were separately accumulated in the base of the metal enclosure, whose walls were intensely cooled.

In order to test the manufacture of cellular concrete without the cement and aggregates usually used in making traditional building concrete, the modern solution for the mixture, including calcined gypsum, hydrated lime, coal fly ash, perlite, silica fume, carboxymethyl cellulose (CMC), and aluminum powder (as an expanding agent), as well as distilled water, was adopted in the four experimental variants shown in Table 2.

It was experimentally found that there was a correlation with the coal fly ash/perlite/silica fume weight ratio in the starting mixture, which influences the fineness and uniformity of the cellular concrete’s porous structure. The four variants were adopted so that the proportions of coal fly ash increased from 3.6 to 11.1%. Correspondingly, the increase in fly ash content involved a reduction in the perlite proportion from 6.4 to 4.6% and a decrease in the silica fume content from 1.2 to 0.3%. The calcined gypsum content as the main binder of the concrete was reduced from 82.3 to 74.7%, corresponding to its substitution with coal fly ash. Having a role as a foam stabilizer, CMC was used at a constant weight proportion of 0.5% in all tested variants. Hydrated lime, as the supplier of Ca(OH)_2_ in the aqueous solution, which facilitates the corrosion of aluminum powder in order to release hydrogen as a foaming gas for cellular concrete manufacture, was used within the limits of 5.0–7.8%.

The details of the mixture’s preparation process were as follows. The mixing after dosing the solid components (gypsum, lime, coal fly ash, perlite, silica fume, CMC, and aluminum powder) was performed by mechanical stirring. Distilled water was poured over the mixture after its homogenization. During this time, the stirring of the mixture continued. After forming the slurry, it was poured into a metal mold with removable walls and allowed to expand freely. The temperature range at which the preparation of fresh concrete was carried out was 70–80 °C (obtained by placing the mixing vessel inside a pre-heated radiant oven.

### 2.3. Characterization Methods for Concrete Specimen Investigation

The determination of the main physical, thermal, and mechanical features of cellular concrete specimens was performed through the methods shown below. The bulk density was carried out according to PN-EN 12390-7:2011, with the sample being kept for 24 h in a mold whose walls did not absorb the sample moisture [3]. The porosity was calculated as a percentage of the difference between the estimated density of fresh concrete without pores and the measured apparent density, also including the pore volume related to the density of concrete without pores, according to NE 012-99 (Cod, 1999). The thermal conductivity (at 25 °C) was measured based on SR EN ISO 8990:2002 and SR EN 1946-3: 2004 [27] and the compressive strength was measured after steam hardening using typical equipment for ordinary concretes, through which standard cubic samples were pressed axially according to PN-EN 12390-3: 2011. The test for determining the water absorption (ASTM D570-ThorLabs, Tokyo, Japan) was carried out by immersing the hardened concrete sample under water for 24 h. The microstructural appearance of the concrete specimens was identified with an ASONA 100X Zoom Smartphone Digital Microscope- ThorLabs, Tokyo, Japan.

## 3. Results and Discussion

### 3.1. Results

A method close to that used in the current work for making cellular concrete via foaming with aluminum powder was recently experimentally applied to produce geopolymeric lightweight concrete using aluminum powder in sodium silicate solution [19]. The procedure was continued with steam curing at 40 °C. The results showed that the duration of the hardening process was significantly reduced, the concrete strength values increased by 80–90%, and the thermal conductivity decreased by up to 0.22 W/mK.

The technique adopted by the paper’s authors for curing the fresh cellular concrete with steam at 0.3 bar and 75 °C being periodically blown for 3.5 h of a total of 8 h to keep the concrete samples at 80 °C in a sealed enclosure (see Section 2.1) influenced the characteristics of the experimental specimens, with the results being presented in Table 3.

The characteristics of the cellular concrete samples made under the technical conditions presented in the paper corresponded to the adopted objectives. Thus, the bulk density values were within the estimated limits (609–641 kg/m^3^). The relatively low density together with the thermal conductivity over a range of low values (0.129–0.144 W/mK) contributed decisively to the heat-insulating properties of the cellular concrete. In a relationship of inverse proportionality to these properties, the concrete porosity increased with the decreases in density and thermal conductivity values from 74.2 to 75.9%. The main mechanical characteristic (compressive strength) simultaneously increased with the increases in bulk density and thermal conductivity, reaching a maximum value of 4.1 MPa in the case of the specimen corresponding to variant 1, largely due to the minimal content of coal fly ash and the maximal contents of perlite and silica fume, respectively, which allowed a homogeneous structure with small pores to be obtained. Regarding the water absorption in the concrete mass, the values varied between 2.6 and 3.5 vol.%. According to the previous literature observations, a material in which the volume of the voids (pores) is smaller and the volume of the ceramic solid mass is higher absorbs more moisture compared to a similar material containing more voids. Thus, the volume of water absorbed by the specimen corresponding to variant 1 reached the maximum value of 3.5 vol.% under the conditions in which this sample had the highest bulk density and the lowest porosity.

Unlike the first three variants tested, the macrostructural aspect of variant 4, during the making of which the minimum proportion of calcined gypsum (74.7%) and the maximum content of coal fly ash (11.1%) were used, showed a relatively inhomogeneous pore distribution in the cross-section. This physical characteristic led to a decrease in compressive strength by almost 50% compared to the concrete specimen corresponding to variant 1, although the bulk density and thermal conductivity values remained at a low level.

**Figure 2:**
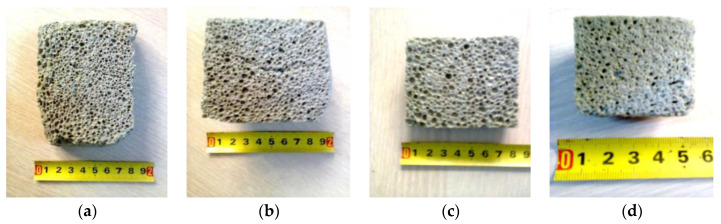
Cross-section appearance of cellular concrete specimens: (**a**) variant 1; (**b**) variant 2; (**c**) variant 3; (**d**) variant 4.

The cross-section appearance of the cellular concrete specimens is shown in Figure 2.

**Figure 3:**
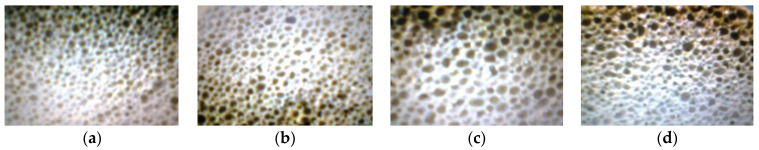
Microstructural images of cellular concrete specimens: (**a**) variant 1; (**b**) variant 2; (**c**) variant 3; (**d**) variant 4.

The microstructural homogeneity of the cellular concrete specimens was investigated by examining the pictures shown in Figure 3. The microstructural appearance of the samples (Figure 3a–c) shows their fine and uniform porosity, with the dimensions increasing from 0.2–1.0 mm (Figure 3a) to 0.5–1.6 mm (Figure 3c). As mentioned above, one specimen (Figure 3d) was characterized by an obvious non-uniformity of the pore distribution, with extremely small pores of 0.1 mm being mixed with much larger pores reaching up to 1.5 mm.

### 3.2. Discussion

The major advantage of cellular concrete is its light weight, which saves the support structures of the building (foundations and walls), meaning high degree of thermal and acoustic insulation is achieved. Additionally, due to the porous structure, the cellular concrete ensures the important economy of material used in the manufacturing process.

Together with hydrogen peroxide (H_2_O_2_) and calcium carbide (CaC_2_), the aluminum powder has the ability to generate a porous structure in the concrete, releasing hydrogen, oxygen, and acetylene. Among these, the use of aluminum powder is the most often applied as an aerating agent [12]. In addition to the manufacture of cellular concrete, it has also been experimentally demonstrated in other foaming processes using liquid agents or agents that are soluble in aqueous solutions and for which their liquid state allows a much more intimate contact between the material subjected to foaming and the agent, leading to fine porous structures being obtained.

Generally, there is a correlation between the density of a cellular concrete and its compressive strength, with the two characteristics increasing simultaneously by decreasing the total volume of the internal voids (pores). The interest of concrete manufacturers is to obtain high mechanical strength under conditions where the density is kept at a low level. This goal in terms of quality became achievable by applying the steam curing method.

## 4. Conclusions

The objective of the work was making a high-strength cellular concrete with a relatively low density. The main components of traditional concrete (cement and aggregate) were not used in this experiment. Calcined gypsum (CaSO_4_·0.5H_2_O) was the main binder of the mixture, with adequate physical, thermal, and ecological properties for building concretes. Coal fly ash was introduced into the mixture due to its contribution to improving the properties of the concrete. The inorganic filler perlite was adopted for its ability to reduce the pore size. Due to the pozzolanic properties, silica fume was also added into the mixture. The adopted foaming agent was aluminum powder prepared using the authors’ original method from aluminum waste melted via unconventional microwave heating. The nitrogen distributed in converging jets upon contact with the molten aluminum jet allowed the atomization of the melt and its capture in a metal enclosure with water-cooled walls in the form of particles with grain sizes below 10 μm. Hydrated lime Ca(OH)_2_ dissolved in distilled water provided the aqueous solution that facilitated the corrosion reaction of the fine aluminum particles with the release of hydrogen as an expansion gas. The fresh concrete was cured by periodical blowing steam at 0.3 bar and 75 °C under laboratory conditions in a room that was permanently heated to 80 °C for 8 h, of which the steam blowing last for 3.5 h. Of the four variants tested, variant 1, in which the weight proportions of the mixture components were 82.3% calcined gypsum, 5% hydrated lime, 3.6% coal fly ash, 6.4% perlite, 1.2% silica fume, 0.5% CMC, 1% aluminum, and 41.6% water, was considered optimal. The main experimentally identified characteristics of the cellular concrete were its bulk density of 641 kg/m^3^, porosity of 74.2%, thermal conductivity of 0.144 W/mK, compressive strength of 4.1 MPa, water absorption rate of 3.5 vol.%, and pore size range of 0.2∓1 mm. In comparison with the data from the literature, the characteristics of the experimentally produced cellular concrete were excellent, the concrete being suitable for building applications.

## Figures and Tables

**Table 1:** The oxide compositions of coal fly ash, perlite, and silica fume.

Composition	Coal Fly Ash (wt.%)	Perlite (wt.%)	Silica Fume (wt.%)
SiO_2_	46.5	70–75	85–98
Al_2_O_3_	23.7	12–15	<2.0
Na_2_O	-	3–4	3–5
K_2_O	10.1	<1.8	<1.1
Fe_2_O_3_	8.6	0.5–2	<1.8
MgO	1.2	0.2–0.7	<1.9
CaO	7.9	0.5–1	<2.5
Other oxides	-	-	<4.0

**Table 2:** The experimental variants.

Composition	Variant (wt.%)			
	1	2	0.3	4
Calcined gypsum	82.3	81.2	78.4	74.7
Hydrated lime	5.0	5.6	7.4	7.8
Coal fly ash	3.6	4.8	6.9	11.1
Perlite	6.4	5.9	5.2	4.6
Silica fume	1.2	1.0	0.6	0.3
CMC	0.5	0.5	0.5	0.5
Aluminum powder	1.0	1.0	1.0	1.0
Distilled water	41.6	41.0	39.7	37.8

**Table 3:** The physical, thermal, and mechanical properties of the samples.

Feature	Variant			
	1	2	3	4
Bulk density (kg/m^3^)	641	625	609	612
Porosity (%)	74.2	75.0	75.9	75.7
Thermal conductivity (W/mK)	0.144	0.135	0.129	0.130
Compressive strength (MPa)	4.1	3.7	3.3	2.3
Water absorption (vol.%)	3.5	3.2	2.6	2.8
Pore size (mm)	0.2–1.0	0.3–1.2	0.5–1.6	0.1–1.5

## Data Availability

Not applicable.
